# Determinants of self rated health and mortality in Russia – are they the same?

**DOI:** 10.1186/1475-9276-7-19

**Published:** 2008-07-25

**Authors:** Francesca Perlman, Martin Bobak

**Affiliations:** 1 ECOHOST, London School of Hygiene and Tropical Medicine, London, UK; 2 Department of Epidemiology and Public Health, University College London, London, UK

## Abstract

**Background:**

Research into Russia's health crisis during the 1990s includes studies of both mortality and self-rated health, assuming that the determinants of the two are the same. In this paper, we tested this assumption, using data from a single study on both outcomes and socioeconomic, lifestyle and psychological predictor variables.

**Methods:**

We analysed data from 7 rounds (1994–2001) of the Russia Longitudinal Monitoring Survey, a panel study of a general population sample (11,482 adults aged over 18 living in households of 2 or more people). Self-rated health was measured on a 5 point scale and dichotomised by combining responses "very poor" and "poor" into poor health. Deaths (n = 782) during a mean follow up of 4.1 years were reported by another household member. Associations between several predictor variables and poor or very poor self-rated health and mortality were measured using logistic regression and Cox proportional hazards analysis respectively.

**Results:**

Poor self-rated health was significantly associated with mortality; hazard ratios, compared with very good, good or average health, were 1.69 (1.36-2.10) in men and 1.74 (1.38-2.20) in women. Low education predicted both mortality and poor self-rated health, but income predicted subjective health more strongly. Smoking doubled the risk of death but was unrelated to subjective wellbeing. Frequent drinkers experienced greater mortality than occasional drinkers, despite reporting better health. In contrast, dissatisfaction with life predicted poor self-rated health, but not mortality.

**Conclusion:**

Differences between the predictors of subjective health and mortality, even though these outcomes were strongly associated, suggest that influences on subjective health are not restricted to serious disease. These findings also suggest the  presence of risk factors for relatively sudden deaths in apparently well people, although further research is required. Meanwhile, caution is required when using studies of self-rated health in Russia to understand the determinants of mortality.

## Background

Life expectancy in Russia stagnated during the 1960s, and then lagged progressively further behind the rising longevity of the countries of Western Europe [1]. After a brief improvement in life expectancy in Russia during the 1980s, the fall of Communism in 1991 heralded an unprecedented further decline [2], that became known as Russia's "mortality crisis". During the transition, fluctuations in mortality followed changes in macroeconomic measures, such as GDP [3], and the greatest proportion of excess deaths was amongst middle-aged men and the least educated [4,5].

Research into the determinants of mortality in Russia to date includes very few prospective studies [4,6]. Studies using other designs, such as case-control studies [7], indirect methods using widowhood [8] or sibling [9] data, and population level studies based on census information [10], have identified education [4,6,9,10], alcohol [9,11], marital status [6] and smoking [9] as important determinants of mortality.

Cross-sectional surveys of the determinants of self-rated health [12,13] have also been used in an attempt to cast light on the causes of ill health in Russia. Self-rated health predicts death consistently in many countries [14], with worsening subjective health associated with progressively higher mortality [15], chronic disease and behavioural risk factors [16]. As a measure, self-rated health performs well: it is stable, with good test-retest reliability, and consistent reporting [14]. The relationship between self-rated health and mortality is surprisingly consistent between countries, despite international variations in the average health state [14].

There is therefore good reason to believe that the predictors of subjective health and mortality are likely to be similar.

Cross-sectional studies have linked self-rated health strongly to material measures, including subjective economic difficulties [17,18], and amongst psychosocial measures, perceived control predicted self-rated health in two studies [12,17]. However, associations between self-rated health and alcohol [18], smoking [19] and education [17] in Russia were less consistent.

Two particular issues in post-transition Russia are of especial relevance to the association between self-rated health and mortality. First, subjective health is worse than in many other countries [20,21] and, since individual level mortality data in Russia is in limited supply, self-rated health has previously been used as a substitute for mortality. Second, the rapid rise in deaths since the transition, together with major fluctuations in life expectancy [22] and a high frequency of sudden deaths [23], including an excess of sudden cardiac and external cause mortality [2,10], could suggest that many deaths are not preceded by prolonged illness, or even a gradual decline in health. However, to our knowledge, the assumption that the determinants of the two are similar has not been tested.

In this paper, therefore, we aim to test the association between self-rated health and mortality in Russia, to compare the associations of different predictor variables (socioeconomic, lifestyle and psychological) with these two measures, and to consider possible explanations for the findings.

## Methods

### Data

The data were from 7 rounds (1994–2002) of the second phase of the Russia Longitudinal Monitoring Survey (RLMS: ), a panel study of households and the individuals within them from 38 population centres across the Russian Federation. Of these, St Petersburg and Moscow were selected automatically, and the remainder were sampled by stratifying according to socioeconomic criteria. Districts were selected from each stratum using a probability proportional to size (PPS) sampling method (where the likelihood of selection was proportional to its population size). Within the selected districts, smaller urban and rural areas were selected, again using PPS, using census enumeration districts and villages respectively. Ten households were selected from each urban area from housing lists developed by the investigators (official housing lists were considered unreliable), and from each rural area using village housing lists. The first dwelling was chosen at random, and the remainder at regular intervals. The average response rate in RLMS was 84% in the first round of Phase 2, and 80% in the second round, although it was lower in Moscow and St Petersburg (67%). New households were recruited in each round to replace those that left the study.

Datasets from each of the seven study rounds were combined. Individuals were included in the final data analysis if their sex and date of birth matched in each dataset. We omitted people who lived alone, since there was no-one to report their deaths. Baseline measurements were taken at the time an individual entered the study.

### Measurements

#### Health variables

##### Self-rated health

Answers to the question "how would you evaluate your health?" were graded on a 5-point scale, similar to many other studies [14]: very good; good; average, poor; or very poor. For some of the later analyses, self-rated health was dichotomised into poor or very poor, versus very good, good or average.

##### Mortality

In each round, respondents were asked about household members from the previous round who had moved away, or had died. The latter group formed the recorded deaths in our analyses. A total of 782 deaths were reported over a mean follow up period of 4.1 years. The death rates in RLMS were similar to national Russian mortality data [24,25].

### Socioeconomic position

*Education *was divided into complete higher; complete secondary; (any of the 3 types of secondary education in Russia: technical, general or combined); and incomplete secondary or primary.

*Household income *Monthly household income was adjusted to the value of the 1992 rouble, to compensate for inflation and currency devaluation. Income from all sources was calculated by the investigators, including earnings, benefits, selling goods and loans and donations from friends, family and other sources. Household income per person was calculated by dividing the total household income by the square root of the number of household members [26], and then dividing into quintiles.

### Health behaviours

Frequency of alcohol consumption was divided into the following 5 categories: none in the last month; once in the last month; 2–3 times per month; once a week; and more than once a week. Respondents were classified as current smokers or non-smokers (either ex-smokers or never-smokers).

### Psychological variables

*Life satisfaction *was ascertained by asking "to what extent are you satisfied with your life in general at the present time?". Responses were graded on a 5-point scale: fully satisfied, rather satisfied, both yes and no, less than satisfied, and not at all satisfied.

### Statistical analysis

First, we examined the distribution of self-rated health in the study population at the time respondents entered the study. Second, we estimated the association between self-rated health and mortality, separately for men and women, using Cox proportional hazards analysis, with self-rated health as both a 5-point ordinal scale and as a dichotomous variable (poor or very poor vs. very good, good or average). We used four multivariate models, adjusting for (i) age; (ii) age, alcohol and smoking; (iii) age, satisfaction and optimism; and (iv) age, income and education. Each model was adjusted for clustering of subjects in households, using the "cluster" sub-command in Stata 9.

On the basis of findings from the literature, we then selected the predictor variables for the main analyses. Education [4], smoking [9] and alcohol [2] are known predictors of mortality in Russia; education and material measures are known to be associated with self-rated health [12]. Life satisfaction can serve as a marker for negative affect and could thus be used to assess any reporting bias in self-rated health, compared to the more objective measure of mortality. The associations between these predictor variables and mortality and poor or very poor self-rated health were then estimated using Cox proportional hazards analysis and logistic regression, respectively. Again, all analyses were adjusted for age and clustering by household. We additionally adjusted for four broad regions of Russia (Central, Ural and the North; Moscow & St Petersburg; Volga and the North Caucasus; and Siberia and the Far East).

## Results

More than half the respondents rated their health as "average". Eleven percent of men and 22% of women described their health as poor or very poor. Few people placed themselves in the categories at the extreme ends of the scale (Table 1). Nearly half the respondents had a university education. More than half the male respondents smoked, and most consumed alcohol regularly. In contrast, far fewer female respondents smoked or drank. More than half the study sample was dissatisfied or very dissatisfied with their lives (Table 1).

**Table 1:** Distribution of self-rated health and other predictor variables

	**Male – mean(SD)**	**Female – mean (SD)**
**Age**, mean *(SD) *	40.6 *(15.6)*	43.3 *(17.9)*
**Household income per person**		
Household income per capita, mean (SD), 1992 roubles	4855 *(8307)*	4698 *(7831)*

	**Male – number (%)**	**Female – number (%)**

**Self-rated health**		
Very good	162 (3.2)	67 (1.1)
Good	1801 (35.2)	1236 (20.1)
Average	2575 (50.3)	3596 (58.5)
Poor	498 (9.7)	1038 (16.9)
Very poor	82 (1.6)	215 (3.5)
*Number of respondents*	*5118*	*6152*
**Deaths**	452 (8.6)	330 (5.3)
	*5,238*	*6,244*
**Education**		
Primary or incomplete secondary	1051 (20.1)	1213 (19.5)
Complete secondary (general and/or technical)	2083 (39.8)	1747 (28.0)
Higher	2095 (40.1)	3272 (52.5)
*Number of respondents*	*5229*	*6232*
**Smoking**		
Current smoker	3202 (62.0)	822 (13.0)
Never smoked	1001 (19.5)	4878 (79.1)
Ex smoker	935 (18.2)	468 (7.6)
*Number of respondents*	*5138*	*6168*
**Frequency of alcohol intake**		
Once in last month	645 (12.0)	1164 (19.0)
2–3 times in last month	1203 (23.3)	1086 (17.5)
Once/week	981 (19.0)	452 (7.3)
More than once a week	965 (18.7)	214 (3.5)
No alcohol	1372 (27.0)	3289 (53.0)
*Number of respondents*	*5166*	*6205*
**Life satisfaction**		
Very satisfied	254 (4.9)	207 (3.4)
Rather satisfied	723 (14.0)	749 (12.1)
Yes and no	1145 (22.0)	1322 (21.4)
Rather dissatisfied	1954 (37.7)	2410 (39.0)
Very dissatisfied	1105 (21.0)	1490 (24.1)
*Number of respondents*	*5181*	*6178*

In men there was a clear mortality gradient across all five categories of self-rated health, where worse health predicted higher mortality (Table 2). In women, however, this gradient existed only between average, poor and very poor health. Women with good health had higher mortality than those with average health, and too few women reported very good health to analyse the association. Using the dichotomised measure, however, men and women with poor or very poor health had significantly higher mortality than those who did not. The association between self-rated health and mortality was not explained by health behaviours, satisfaction and optimism or by income and education. The fully adjusted hazard ratios were 1.69 (1.36–2.10) in men and 1.74 (1.38–2.20) in women.

**Table 2:** Relationship between self-rated health (as a continuous and dichotomous) variable and mortality

	**Cox proportional hazards ratio for death during the study** ** (95% confidence interval)**	
	**Male**	**Female**

**Self-rated health at entry**		

***(a) Ordinal Variable***		
Very good	0.62 (0.23–1.68)	-
Good	0.84 (0.64–1.11)	**1.73 (1.08–2.77)**
Average	1	1
Poor	**1.52 (1.20–1.93)**	**1.66 (1.29–2.14)**
Very poor	**2.40 (1.66–3.46)**	**2.49 (1.77–3.48)**
*P for trend*	*<0.001*	*<0.001*

***(b) Dichotomous variable***		
Very good/good/average	1	1
Poor/very poor	**1.69 (1.36–2.10)**	**1.74 (1.38–2.20)**

The associations between predictor variables, self-rated health and mortality are shown in Table 3. Men and women with less education experienced significantly higher mortality and, to a slightly lesser degree, worse self-rated health. There was a significant trend in the association between household income and self-rated health, particularly in men. In contrast, the association with mortality was non-significant. Smoking was significantly associated with mortality in both sexes but not with self-rated health. People who consumed alcohol 2–3 times a month or once a week, compared with once a month, had significantly better self-rated health but, in contrast, drinking more than once a week was associated with elevated mortality compared with monthly alcohol consumption in both sexes, although it had no significant association with subjective health. Abstention, compared with consuming alcohol once a month, significantly predicted both mortality and poor self-rated health in both men and women. Greater life satisfaction was associated with significantly better self-rated health but not with mortality. There was no statistically significant interaction between self-rated health and either education or income in its association with mortality in either sex.

## Discussion

**Table 3:** Association between socioeconomic and other measures and self-rated health at entry (logistic regression analysis) and mortality (Cox proportional hazards analysis)

	**Male**		**Female**	
	**Odds ratio for poor or very poor self-rated health at entry**	**Hazard ratio (95% confidence interval) for death during study)**	**Odds ratio for poor or very poor self-rated health at entry**	**Hazard ratio (95% confidence interval) for death during study)**

**Education**				
Primary/incomplete secondary	1.70 (1.35–2.12)	2.30 (1.85–2.86)	1.32 (1.11–1.58)	3.09 (2.39–4.00)
Complete secondary	1.27 (0.99–1.63)	1.39 (1.09–1.78)	1.03 (0.86–1.24)	2.35 (1.63–3.40)
Higher	1	1	1	1
Continuous (per 1 category)	1.30 (1.16–1.45)	1.52 (1.36–1.70)	1.14 (1.04–1.24)	1.74 (1.54–1.97)

**Household income per person**				
(household income/square root no. in household)				
1 (lowest quintile)	1.64 (1.21–2.22)	1.41 (1.05–1.88)	1.34 (1.06–1.70)	1.19 (0.86–1.65)
2	1.21 (0.90–1.63)	0.98 (0.73–1.30)	1.20 (0.96–1.51)	0.94 (0.67–1.30)
3	1	1	1	1
4	1.06 (0.79–1.44)	0.89 (0.66–1.21)	0.99 (0.78–1.26)	1.00 (0.72–1.39)
5 (highest quintile)	0.68 (0.49–0.95)	1.12 (0.83–1.52)	0.87 (0.67–1.11)	0.75 (0.50–1.13)
Change per quintile	0.83 (0.77–0.89)	0.94 (0.88–1.02)	0.90 (0.85–0.95)	0.93 (0.85–1.01)

**Alcohol frequency**				
No alcohol	1.84 (1.38–2.47)	1.42 (1.05–1.93)	1.42 (1.17–1.71)	2.00 (1.34–3.00)
Once in last month	1	1	1	1
2–3 times a month	0.66 (0.47–0.94)	1.02 (0.72–1.46)	0.75 (0.57–0.98)	1.27 (0.71–2.27)
Once a week	0.54 (0.37–0.80)	1.10 (0.77–1.56)	0.63 (0.42–0.94)	1.08 (0.47–2.52)
More than once a week	0.83 (0.58–1.17)	1.93 (1.41–2.65)	0.92 (0.57–1.50)	2.46 (0.97–6.24)

**Current smoker**				
Yes (vs no)	0.98 (0.81–1.19)	1.92 (1.58–2.34)	1.17 (0.90–1.50)	2.77 (1.68–4.56)

Life satisfaction (5 point ordinal scale, high to low)	1.40 (1.27–1.54)	0.99 (0.91–1.08)	1.46 (1.35–1.58)	1.08 (0.98–1.19)

**Income and education **Education (3 cats), household income per person (household income/square root no. in household) quintile **N.B. All models adjust for age at entry and cluster by household**

### Summary of key findings

In this large Russian population-based study, the majority of respondents of either sex rated their health as "average", the middle of 5 categories, and 11% of men and 20% of women reported poor or very poor health. Few respondents placed themselves at the extreme ends of the scale. Self-rated health was significantly associated with mortality, to a similar degree in both sexes. There were important differences in the associations between several predictor variables and poor or very poor self-rated health and mortality. Education was associated with both outcomes, although more strongly with mortality. Smoking was predicted mortality, but not self-rated health. Household income, life satisfaction and optimism were positively associated with self-rated health, but not with mortality. Frequent alcohol consumption was linked to better self-rated health but, conversely, to higher mortality.

#### Strengths and weaknesses

RLMS is probably the largest population study from post-transition Russia to date, and provides a unique opportunity to study the determinants of health at a time of major social change and profound mortality crisis. The study sample was representative of the national population in terms of age, sex and geography, and the findings are therefore likely to be generalisable across Russia. The findings are supported further by the generally high response rate and the relatively low proportion of respondents with missing data (less than 0.5% for education and area of residence, less than 2% for smoking and alcohol consumption, and 6% for household income).

The study had several potential limitations. First, deaths were reported by the relatives and could have therefore been under-reported. However, mortality rates were similar to the national figures [27], and the association between education and mortality [4,10], and smoking and mortality [9] were in line with other studies suggesting the absence of any major bias in mortality ascertainment.

Second, twenty-five percent of individuals left RLMS without explanation, and these were more likely to be young, less educated, urban residents with higher incomes. People living alone, who were excluded because of the risks of under-reporting of mortality, were older and less affluent than other respondents. The study sample may thus not be fully representative for the national population but the similarity of the associations between education, smoking and mortality here and in other studies [4,9,10] indicates good internal validity of the findings.

Third, difficulties in measuring income may have led to underestimation, as a result of inflation, wage arrears [28], and widespread non-monetary earnings (e.g. bartering or exchanging of favours). However, more detailed analyses elsewhere showed that material (non-monetary) factors did not predict mortality strongly [24,25].

Fourth, alcohol consumption was not measured by individual type, and some alcohols, such as surrogate alcohols which are widely consumed, appear to predict mortality particularly strongly [7].

Finally, variations in factors between years could have influenced the results, since baseline data was taken from the year of entry into RLMS, and subjects entered in different years, although financial measures were adjusted for inflation. Two observations suggest that this effect is not likely to have been important, though. First, the prevalence of poor or very poor self-rated health in the study population varied very little between years (from 17% in 1994 and 1996 to 20% in 1995 and 1998); and second, adjusting the analyses for year of entry did not greatly affect the final results (data not shown).

Finally, using self-rated health a dichotomous measure, rather than utilising the full 5-point scale, could have led to the loss of some information. However, both measures were strongly and significantly associated with mortality, which suggests that any information loss was likely to have been small.

#### Interpretation of the results

In RLMS, more than half the respondents reported "average" self-rated health, similar to the 1996 New Russia Barometer survey (45% in men and 49% in women) [29], and Nicholson et al (50% of both sexes) [30]. However, there were some differences between the Russian studies: fewer respondents in RLMS reported "poor" or "very poor" health than in either the NRB (17% of men and 29% of women) [29], or in Nicholson et al (30% of men and 40% of women) [30]. The prevalence of fair and poor subjective health in RLMS were almost identical to 2 rounds of the World Values Survey before the transition (1981 and 1990) [31]. Self-rated health all the Russian studies was far worse than in English speaking countries where it is typically "good", although somewhat better than the "poor" health [14] typically reported in one Lithuanian study.

The strong, independent association between self-rated health and mortality in RLMS is consistent with studies in other countries [14], and the progressive relationship between worsening self-rated health and increased risks of mortality in men was similar to findings elsewhere [15]. The relationship was less evenly graduated in women, but this could be due to their low prevalence of good and very good health.

Considering these findings in the light of the Russia's rapid fluctuations in mortality as well as theoretical mechanisms proposed for the association between self-rated health and mortality [14], may cast further light on these findings.

A key theory is that that self-rated health influences mortality because it is an "inclusive" measure that summarises objective health status [14,32]. The strong association between self-rated health and mortality in this study and the similarity of the relationships between education and both outcomes support this notion The high prevalence of poor subjective health in Russia may simply indicate high rates of disease, reflected in the low national life expectancy [3].

However, three findings suggest that serious illness is not the only influence on subjective health, and that other factors limit the explanation of mortality by health. First, international differences in the prevalence of self-rated health do not always follow national mortality rates. Self rated health was worse in Lithuania [14] than in this and other Russian studies [29,30], despite Lithuania's higher life expectancy [22]. Similarly, within India, the regions with the worst self-rated health do not experience the highest mortality [33]. Cultural differences in reporting may offer a partial explanation, but are unlikely to account for the second, and related, finding that the prevalence of subjective health in Russia has not changed during the transition [31], despite major fluctuations in life expectancy [2,3]. This could perhaps reflect the high frequency of sudden deaths in Russia [23] where the two commonest causes of premature mortality, cardiovascular disease (which includes sudden cardiac deaths) and external causes, may lead to unexpected deaths in apparently fit individuals who do not perceive themselves as ill [2,10].

Third, important individual level differences in the predictors of self-rated health and mortality also suggest that there were other factors at play. For example, the association between satisfaction and self-rated health, but not mortality, may indicate that psychological factors influence subjective ratings of health. The same was true of income, and although the connection between material measures and health is less clear, some positive emotion may possibly be associated with both. Although this range of variables was limited, such findings are consistent with another study where perceived stress predicted subjective symptoms of angina, but not the objective electrocardiographic signs of heart disease [34].

In contrast, although smoking and frequent alcohol consumption predicted mortality as expected [9,11], neither led to worse subjective health. In fact, somewhat paradoxically, frequent drinkers reported better health than more moderate consumers. This is the first time that better health has been demonstrated amongst Russians with risky health lifestyles individuals in a single, individual-level study. These findings are consistent with gender differences in health in Russia, where men, whose life expectancy [2] and health behaviours [19,35] are worse than women, have better [17] or similar self-rated health [30].

There are at least two plausible explanations for these findings. First, Russians are known to drink to "help them forget everyday cares and difficulties" [36], and whilst they may forget their health concerns, the adverse physical consequences of heavy alcohol consumption remain. Second, some smoking and alcohol related deaths may not have been preceded by ill-health. As discussed above, rapid onset deaths, notably from cardiovascular disease and external causes [2,10,23], are common, and alcohol has been implicated in sudden death [23]. Although the role of smoking is less clear, it could act as a background risk factor for cardiovascular disease.

Abstainers from alcohol, as well as frequent drinkers, were at higher risk of death, consistent with the J-shaped curve seen elsewhere [37], but in contrast non-drinkers also experienced worse self-rated health than moderate consumers. Although the reasons are not clear, as elsewhere, some may previously have been heavy users of alcohol who were now too ill to drink [38].

Two further theoretical mechanisms linking self-rated health to mortality were considered, but these were not supported by our findings. The first was that poor health leads to more risky behaviours, which themselves lead to premature death [14]. However, as noted above, self-rated health and health behaviours were not closely associated here. The second mechanism was that self-rated health acts as a marker of socioeconomic circumstances, and the latter influence mortality [14]. Again, however, these data did not support this mechanism, since subjective health predicted mortality independently of income and education, and it did not interact significantly with either variable here or in another study [39].

In summary, although self-rated health predicted mortality as expected [14], differences in the associations with different predictor variables indicate that subjective evaluations of health may incorporate psychological and other influences in addition to subsequently fatal illness, whereas some health-damaging factors such as smoking and drinking are not considered. Frequent sudden deaths amongst apparently healthy individuals, some of whom had significant behavioural and other risk factors,, may partly explain these differences, and could also explain why self-rated health remained unchanged during Russia's "mortality crisis". These data offered only limited support for several theoretical mechanisms for the influence of self-rated health on mortality.

Further research should study individual causes of death, and a range of health outcomes, for example chronic disease [15,16]. International comparisons would be help to identify any systematic variations the associations shown in this study, for example in relation to rates of sudden death. In the short-term, however, our results indicate that, at least in Russia, extrapolating the predictors of self-rated health to those of mortality should be done with caution.
